# 

**Published:** 1843-05-13

**Authors:** 


CONTENTS.

A Course of Clinical Lectures, delivered at the Hôtel Dieu, Paris, for the Session 1842–'43. By A. F. Chomel, M.D. Lecture 7. Hysteria — Ramollissement, . . . . . . . 97

Quarterly Report of the Obstetric Department of the Philadelphia Dispensary, for First, Second and Third months, 1843, by Joseph Wurrington, Obstetric Physician, . . . . . . 99

*University of Pennsylvania.*

Clinic of Dr. W. Poyntell Johnston.

Case 1.—Varicose Veins of the left Leg, 99

Case 2.—Polypus of the Nose, removed with the forceps, . . . . 103

*Jefferson Medical College.*

Clinic of Professor Mütter.

Case.—Gelatinous Polypus of the Nose—operation with forceps, . . 104

*Bibliographical Notice.*

A Practical and Theoretical Treatise on the Diagnosis, Pathology, and Treatment of Diseases of the Skin, arranged according to a Natural System of Classification, and preceded by an outline of the Anatomy and Physiology of the Skin. By Erasmus Wilson, Lecturer on Anatomy and Physiology, &c. . 104

EDITORIAL—MEDICAL OFFICERS OF THE NAVY, . . . . . . . 104

*Retrospect of the Medical Sciences.*

Disease of Mitral Valves, . . . 107

Tannin in Hooping Cough, . . . . 107

Ointment of the Extract of Nutgalls, . 108

Indian Hemp, . . . . . . 108

Researches concerning the effects of Arsenic on wool-covered Animals, &c. 108

Naphthaline, . . . . . . 108

Treatment of Salivation, . . . . 108

